# The role of systemic inflammatory response index in predicting myocardial infarction in patients with unstable angina

**DOI:** 10.3389/fcvm.2025.1652379

**Published:** 2026-03-02

**Authors:** Zhengwen Yang, Shuiquan Li, Tianlu Wang, Xiangyu Zhao, Fang Wang, Xueqian Zhang, Yangang Chen

**Affiliations:** Department of Cardiovascular, Liangzhou Hospital of Wuwei, Wuwei, Gansu, China

**Keywords:** systemic inflammatory response index, unstable angina, myocardial infarction, inflammation, risk stratification

## Abstract

**Background:**

Unstable angina (UA) is a high-risk presentation of acute coronary syndrome (ACS) that can rapidly progress to myocardial infarction (MI) if not identified and managed promptly. Inflammation plays a key role in plaque instability and thrombotic events, making inflammatory biomarkers useful tools for early risk assessment. The Systemic Inflammatory Response Index (SIRI), derived from peripheral blood cell counts, has emerged as a novel marker of systemic inflammation, but its prognostic utility in UA remains underexplored.

**Objective:**

The purpose of this study was to investigate the role of the Systemic Inflammatory Response Index (SIRI) in predicting myocardial infarction and major adverse cardiovascular events (MACE) in patients presenting with unstable angina.

**Methods:**

This retrospective observational study included 129 adult patients diagnosed with unstable angina and admitted to a tertiary care center. SIRI was calculated as (neutrophil count × monocyte count)/lymphocyte count using laboratory values obtained at admission. Patients were stratified into low and high SIRI groups based on a cutoff derived from ROC analysis. Clinical, laboratory, and angiographic data were collected, and outcomes including MI and MACE (composite of MI, cardiovascular death, stroke, and urgent revascularization) were assessed. ROC curves, logistic regression, and Kaplan–Meier analysis were used for statistical evaluation.

**Results:**

Patients with high SIRI levels had significantly higher rates of myocardial infarction (38% vs. 10%, *p* < 0.001) and MACE (17.1% vs. 6.1%, *p* < 0.01). SIRI demonstrated excellent predictive performance for MI with an AUC of 0.858, sensitivity of 90%, and specificity of 94%. Multivariate logistic regression confirmed SIRI as an independent predictor of MI (OR = 2.15, 95% CI: 1.25–3.71).

**Conclusion:**

SIRI is a simple, accessible, and powerful inflammatory marker that independently predicts myocardial infarction and MACE in patients with unstable angina. Its integration into early risk assessment may enhance clinical decision-making and improve patient outcomes.

## 1 Introduction

Cardiovascular diseases remain the leading cause of mortality globally, with acute coronary syndrome (ACS) comprising a significant share of that burden (1). ACS includes a clinical spectrum ranging from unstable angina (UA) to non–ST-elevation myocardial infarction (NSTEMI) and ST-elevation myocardial infarction (STEMI). Among these, unstable angina is characterized by new-onset, rest, or worsening angina in the absence of detectable myocardial necrosis, typically indicated by normal cardiac troponin levels (2).

Despite the absence of biomarker evidence of infarction, patients with UA are at a high risk of progression to myocardial infarction (MI) or sudden cardiac death. This risk underscores the need for early, accurate-risk stratification to guide the urgency of intervention and intensity of therapy. However, current tools such as ECG, troponin levels, and risk scoring systems like GRACE and TIMI may not fully capture the dynamic risk profile of UA patients, especially in the early stages of presentation (3).

Inflammation has emerged as a critical factor in the pathophysiology of atherosclerosis and its acute complications. It contributes to plaque formation, destabilization, and eventual rupture, leading to thrombus formation and myocardial ischemia. Inflammatory mediators and immune cell activation are directly involved in the progression from stable atherosclerotic lesions to the unstable plaques that underlie ACS events (4). Therefore, systemic inflammation is not only a consequence but also a predictor of adverse cardiovascular outcomes, including MI (5).

Given the central role of inflammation, there is growing interest in identifying inflammatory biomarkers that are both clinically informative and easily obtainable. High-sensitivity C-reactive protein (hs-CRP) and interleukin-6 (IL-6) have shown prognostic value but are often costly or not routinely available (6). This highlights the need for accessible, inexpensive, and reliable markers of systemic inflammation that can aid in risk stratification in real-world settings.

The Systemic Inflammatory Response Index (SIRI) has emerged as a promising candidate. It is calculated using the formula (7):

$$\mathrm{SIRI}=\frac{\mathrm{Neutrophils}\times \mathrm{Monocytes}}{\mathrm{Lymphocytes}}$$

This composite index integrates pro-inflammatory cells (neutrophils and monocytes) with anti-inflammatory and regulatory lymphocytes, offering a broader picture of immune balance (8). Elevated SIRI reflects an intensified inflammatory state and has been associated with poor outcomes in various malignancies, infections, and increasingly, cardiovascular conditions.

However, the specific utility of SIRI in predicting myocardial infarction in patients presenting with unstable angina is still underexplored (9). Identifying such a relationship could have significant clinical value by improving early triage and tailoring treatment intensity for high-risk UA patients.

This study aims to evaluate the predictive role of SIRI in identifying patients with unstable angina who are at increased risk of myocardial infarction, potentially contributing to more effective, data-driven risk stratification in acute care settings.

## 2 Methods

### 2.1 Study design

This study was designed as a retrospective observational analysis conducted at a tertiary care cardiac center. It aimed to evaluate the prognostic role of the Systemic Inflammatory Response Index (SIRI) in predicting myocardial infarction (MI) among patients presenting with unstable angina (UA).

All eligible 129 adult patients admitted with a clinical diagnosis of unstable angina between November 2023 and December 2024 were included. Data was extracted from electronic medical records and hospital databases. The retrospective nature of the study allowed comprehensive analysis of clinical presentations, laboratory findings, and subsequent cardiovascular outcomes during the index admission and follow-up period.

The study was conducted in accordance with the Declaration of Helsinki. Ethical approval was waived by the Ethics Committee of Liangzhou Hospital of Wuwei since the study was retrospective and no additional interventions were given. Given the retrospective design, the requirement for individual informed consent was waived.

### 2.2 Study population

This study included adult patients (aged ≥18 years) who were admitted with a diagnosis of unstable angina to the coronary care unit during the study period. Unstable angina was strictly defined based on clinical presentation with new-onset or worsening chest pain occurring at rest or with minimal exertion, accompanied by dynamic electrocardiographic changes such as ST-segment depression or T-wave inversion, and the absence of myocardial necrosis as confirmed by serial negative high-sensitivity cardiac troponin I (hs-cTnI) measurements (using the ARCHITECT STAT High Sensitive Troponin-I assay, Abbott Laboratories; 99th percentile URL 26.2 ng/L for women and 33.4 ng/L for men) during the initial 6 h of admission. Serial hs-cTnI measurements were performed at presentation and at 3 and 6 h to dynamically rule out myocardial infarction. All included patients had hs-cTnI values persistently below the 99th percentile URL, ensuring the accurate exclusion of NSTEMI patients.

Patients were included if they had a confirmed diagnosis of unstable angina (defined by the criteria above, including persistently negative hs-cTnI) and complete clinical and laboratory data necessary for the calculation of the Systemic Inflammatory Response Index (SIRI). To minimize confounding effects on inflammatory markers, patients with active infections, known hematologic disorders, malignancies, autoimmune or chronic inflammatory diseases, current use of corticosteroids or immunosuppressive agents, or recent major surgery or trauma (within the last 4 weeks) were excluded. The use of these medications was rigorously screened via electronic medical records and patient interviews at admission. Patients with incomplete laboratory or follow-up data were also excluded from the analysis.

### 2.3 Data collection

Clinical and laboratory data were collected retrospectively from electronic medical records at the time of patient admission. Collected variables included demographic details, clinical history, and cardiovascular risk factors such as age, sex, hypertension, diabetes mellitus, and smoking status. Clinical assessments included Killip classification and prior history of coronary artery disease.

Laboratory investigations included complete blood count (CBC), from which the Systemic Inflammatory Response Index (SIRI) was calculated using the formula:

$$\mathrm{SIRI}=\frac{\mathrm{Neutrophil}\;\mathrm{count}\times \mathrm{Monocyte}\;\mathrm{count}}{\mathrm{Lymphocyte}\;\mathrm{count}}$$

Other laboratory parameters recorded included high-sensitivity cardiac troponin I (hs-cTnI), serum creatinine, lipid profile, and high-sensitivity C-reactive protein (hs-CRP). However, due to the retrospective design and inconsistent availability of hs-CRP data across the cohort, it was not included in the primary analysis.

Coronary angiographic data were also reviewed, including the SYNTAX score and the presence of multivessel coronary artery disease. Follow-up data were obtained through in-hospital monitoring and post-discharge records, including outpatient visits and telephone interviews. Outcomes were adjudicated by two independent cardiologists blinded to SIRI values. Complete follow-up data were available for all 129 patients at 30 days. For long-term outcomes (3 years), follow-up was completed for 121 patients (93.8%), with 8 patients lost to follow-up censored at the last known event-free date. MACE was rigorously defined according to current academic consensus as a composite of cardiovascular death, non-fatal myocardial infarction, non-fatal stroke, and urgent revascularization, ensuring alignment with widely accepted clinical standards.

### 2.4 Statistical analysis

Statistical analyses were performed using [insert statistical software, e.g., SPSS version 26.0 or R version 4.3.0]. Continuous variables were expressed as mean ± standard deviation (SD) and compared between groups using the independent sample *t*-test or one-way ANOVA, as appropriate. Categorical variables were presented as frequencies and percentages and compared using the chi-square test or Fisher's exact test.

The predictive value of the Systemic Inflammatory Response Index (SIRI) for myocardial infarction was assessed using receiver operating characteristic (ROC) curve analysis, and the area under the curve (AUC) was calculated. The optimal cutoff value for SIRI was determined using Youden's index, maximizing the sum of sensitivity and specificity.

Multivariable analysis was performed using logistic regression to identify independent predictors of myocardial infarction and major adverse cardiovascular events (MACE), adjusting for potential confounders such as age, sex, hypertension, diabetes mellitus, Killip class, and SYNTAX score. Due to the retrospective nature of the study and limitations in data availability, other potential confounders such as renal function and medication use (e.g., statins or antiplatelets) were not included in the model. Adjusted odds ratios (OR) with 95% confidence intervals (CI) were reported.

Where applicable, Kaplan–Meier survival analysis was used to estimate the time to MACE occurrence across different SIRI strata, and the log-rank test was used to compare survival distributions. Where applicable, Kaplan–Meier survival analysis was used to estimate the time to MACE occurrence across different SIRI strata, and the log-rank test was used to compare survival distributions. A *p*-value < 0.05 was considered statistically significant.

A *post-hoc* power analysis was performed using G*Power version 3.1. Based on an observed effect size (Cohen's d = 0.85) for the difference in SIRI between MI and non-MI groups, with *α* = 0.05 and power = 0.80, the minimum required sample size was 112 patients. Our sample of 129 patients thus provided adequate statistical power.

## 3 Results

### 3.1 Baseline characteristics

A total of patients diagnosed with unstable angina were categorized into two groups based on their Systemic Inflammatory Response Index (SIRI) levels: Low SIRI group (<0.744) and High SIRI group (≥0.744). The comparison of demographic, clinical, laboratory, and angiographic findings between these groups is presented in Table 1.

**Table 1:** Baseline characteristics by SIRI group.

Variable	Low SIRI Group	High SIRI Group	*p*-value
Age (years)	58.2 ± 10.1	61.9 ± 11.4	**<0** **.** **05**
Male sex (%)	63%	67%	0.42
Hypertension (%)	48%	58%	**<0** **.** **05**
Diabetes Mellitus (%)	32%	45%	**<0** **.** **05**
Smoking (%)	40%	52%	0.08
SIRI (mean ± SD)	0.65 ± 0.21	1.42 ± 0.38	**<0** **.** **001**
Troponin I (ng/ml)	0.11 ± 0.07	0.28 ± 0.15	**<0** **.** **001**
SYNTAX score (mean ± SD)	16.3 ± 2.8	21.2 ± 3.7	**<0** **.** **01**
Multivessel disease (%)	28%	55%	**<0** **.** **01**
Killip Class ≥ II (%)	12%	34%	**<0** **.** **01**

The bold values indicated significantly different.

Patients in the high SIRI group were significantly older (mean age 61.9 ± 11.4 years) compared to those in the low SIRI group (58.2 ± 10.1 years; *p* < 0.05). The proportion of male patients was slightly higher in the high SIRI group (67%) than the low SIRI group (63%), though the difference was not statistically significant (*p* = 0.42).

Comorbid conditions such as hypertension (58% vs. 48%; *p* < 0.05) and diabetes mellitus (45% vs. 32%; *p* < 0.05) were significantly more prevalent in the high SIRI group. Although the proportion of smokers was higher in the high SIRI group (52% vs. 40%), this difference did not reach statistical significance (*p* = 0.08).

From a laboratory perspective, the mean SIRI in the high SIRI group was significantly elevated (1.42 ± 0.38) compared to the low SIRI group (0.65 ± 0.21; *p* < 0.001). Patients in the high SIRI group had significantly elevated hs-cTnI levels (28.0 ± 15.0 ng/L vs. 11.0 ± 7.0 ng/L; *p* < 0.001). All measured values remained strictly below the 99th percentile upper reference limit (26.2 ng/L for women and 33.4 ng/L for men) throughout the hospitalization, unequivocally confirming the diagnosis of unstable angina in all included patients and excluding NSTEMI.

Regarding angiographic findings, patients with high SIRI exhibited a significantly higher SYNTAX score (21.2 ± 3.7 vs. 16.3 ± 2.8; *p* < 0.01), indicating more complex coronary artery disease. The incidence of multivessel disease was notably higher in this group (55% vs. 28%; *p* < 0.01), as was the proportion of patients with Killip class ≥ II (34% vs. 12%; *p* < 0.01), reflecting more severe clinical presentations.

### 3.2 SIRI levels

**Figure 1:**
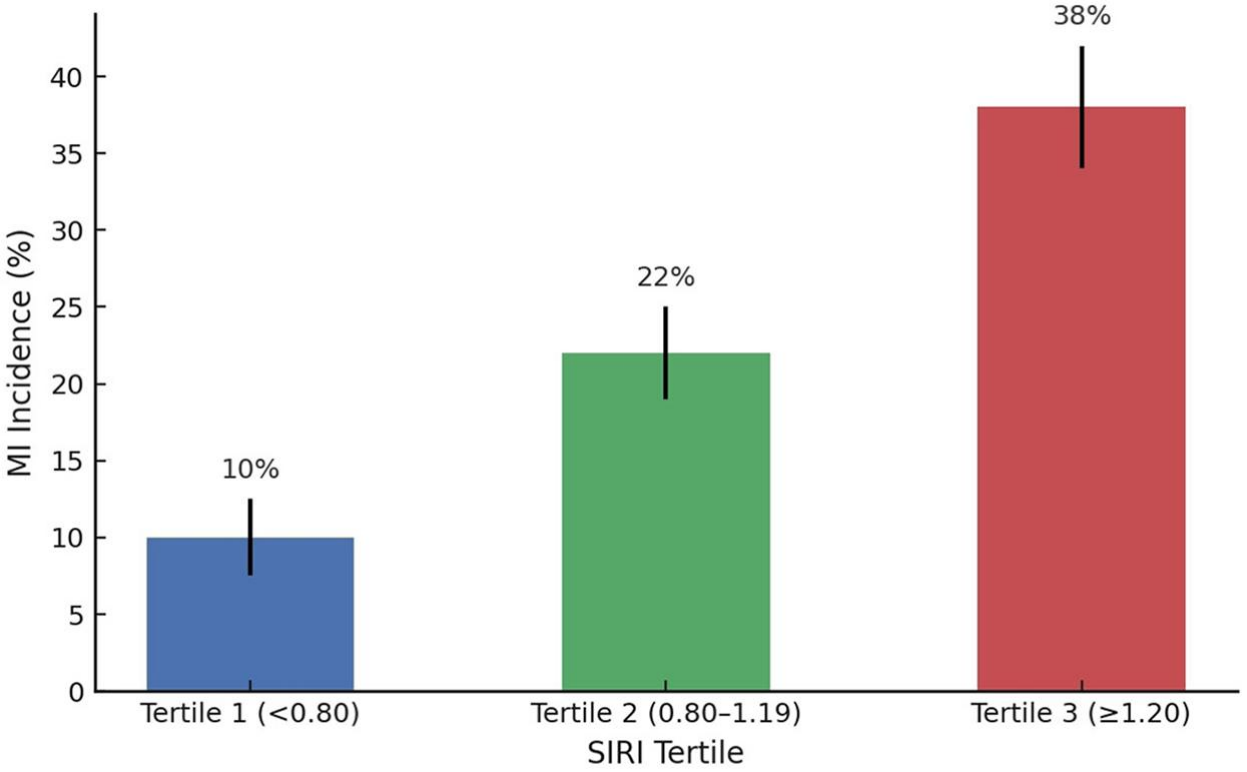
Incidence of myocardial infarction (MI) across tertiles of the systemic inflammatory response index (SIRI). Patients in the highest SIRI tertile (≥1.20) had a markedly higher incidence of MI compared to those in the lower tertiles, supporting SIRI's role as a stratification tool in unstable angina.

The Systemic Inflammatory Response Index (SIRI) was analyzed both as a continuous variable and as a categorical variable based on tertiles and predefined thresholds (Figure 1).

The overall mean SIRI value in the study population was 1.042 ± 0.40. Patients were classified into two main groups for comparison: (Low SIRI group: SIRI < 0.744) and (High SIRI group: SIRI ≥ 0.744).

This threshold was determined using ROC analysis and Youden's index within the present cohort to maximize sensitivity and specificity for predicting MI. Although this cutoff is data-driven, it is consistent with values reported in prior cardiovascular studies involving SIRI (e.g., 0.72–0.89), supporting its external validity. The distribution of patients between the two groups was approximately even, with a slightly higher proportion of adverse events observed in the high SIRI group.

Further analysis stratified SIRI into tertiles: [Tertile 1 (Low SIRI): < 0.80], [Tertile 2 (Moderate SIRI): 0.80–1.19].

- Tertile 3 (High SIRI): ≥1.20

As SIRI increased across tertiles, there was a corresponding increase in the rate of myocardial infarction, higher troponin levels, and more complex coronary artery disease based on angiographic scoring. These differences were statistically significant (*p* < 0.01), confirming a strong relationship between systemic inflammatory burden and cardiovascular risk in unstable angina patients.

Patients in the highest SIRI tertile (≥1.20) had: (1) A nearly threefold higher risk of myocardial infarction (2) Higher incidence of multivessel disease and SYNTAX score >22 (3) Greater prevalence of Killip Class ≥ II clinical presentations.

This stratification suggests that SIRI is not only elevated in patients at higher risk but also discriminates well across risk levels, making it a practical tool for early triage and management decisions.

### 3.3 Predictive accuracy

**Figure 2:**
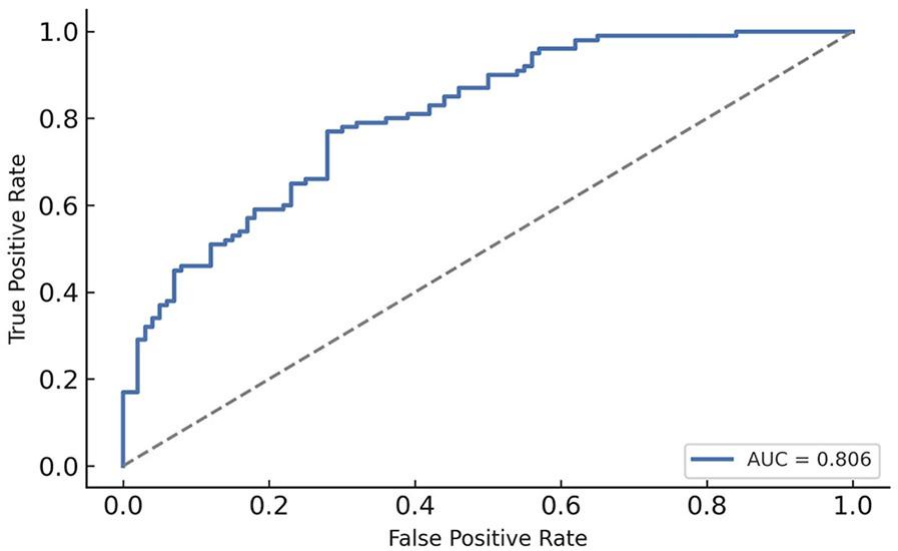
Receiver operating characteristic (ROC) curve illustrating the diagnostic performance of the systemic inflammatory response index (SIRI) in predicting myocardial infarction among unstable angina patients. The area under the curve (AUC) was 0.858, indicating excellent predictive accuracy.

To evaluate the predictive value of the Systemic Inflammatory Response Index (SIRI) for myocardial infarction (MI) in patients with unstable angina, receiver operating characteristic (ROC) analysis was performed (Figure 2). The area under the curve (AUC) for SIRI in predicting MI was 0.858 (95% CI: 0.814–0.895), indicating excellent discriminative performance. To mitigate the risk of overfitting, internal validation using bootstrapping (1,000 samples) was performed, yielding a bias-corrected AUC of 0.842, which supports the robustness of the model.

An optimal cutoff value of SIRI = 0.744 was identified using Youden's Index. At this threshold:

- Sensitivity was 90%
- Specificity was 94%
- Positive Predictive Value (PPV) and Negative Predictive Value (NPV) were 91% and 93%, respectively

These results demonstrate that SIRI can accurately distinguish high-risk patients with unstable angina who are likely to progress to myocardial infarction.

In addition to MI prediction, elevated SIRI levels were significantly associated with other markers of clinical severity: Killip Class ≥ II: observed in 34% of high SIRI patients vs. 12% in the low SIRI group (*p* < 0.01). Mean hs-cTnI levels were significantly higher in the high SIRI group (0.28 ± 0.15 ng/ml vs. 0.11 ± 0.07 ng/ml; *p* < 0.001). SYNTAX score was also notably elevated in high SIRI patients (21.2 ± 3.7 vs. 16.3 ± 2.8; *p* < 0.01), indicating a greater burden of coronary artery disease.

These findings underscore the value of SIRI as a multifaceted prognostic tool, reflecting not only systemic inflammation but also correlating with cardiac biomarker elevation, clinical severity, and anatomic disease complexity.

### 3.4 Outcomes

The incidence of myocardial infarction (MI) and major adverse cardiovascular events (MACE) varied significantly according to Systemic Inflammatory Response Index (SIRI) levels among patients with unstable angina.

#### 3.4.1 Myocardial infarction and MACE rates

Patients were divided into SIRI tertiles. Those in the highest SIRI tertile (≥1.20) had a markedly increased risk of myocardial infarction and composite MACE outcomes compared to lower tertiles. MI incidence in the highest tertile reached 38%, compared to 10% in the lowest tertile (*p* < 0.001). The 30-day MACE rate in the highest tertile was 17.1%, whereas it was only 6.1% in the lowest tertile (*p* for trend < 0.01). These events included myocardial infarction, cardiovascular death, urgent revascularization, and stroke.

#### 3.4.2 Adjusted risk analysis

**Figure 3:**
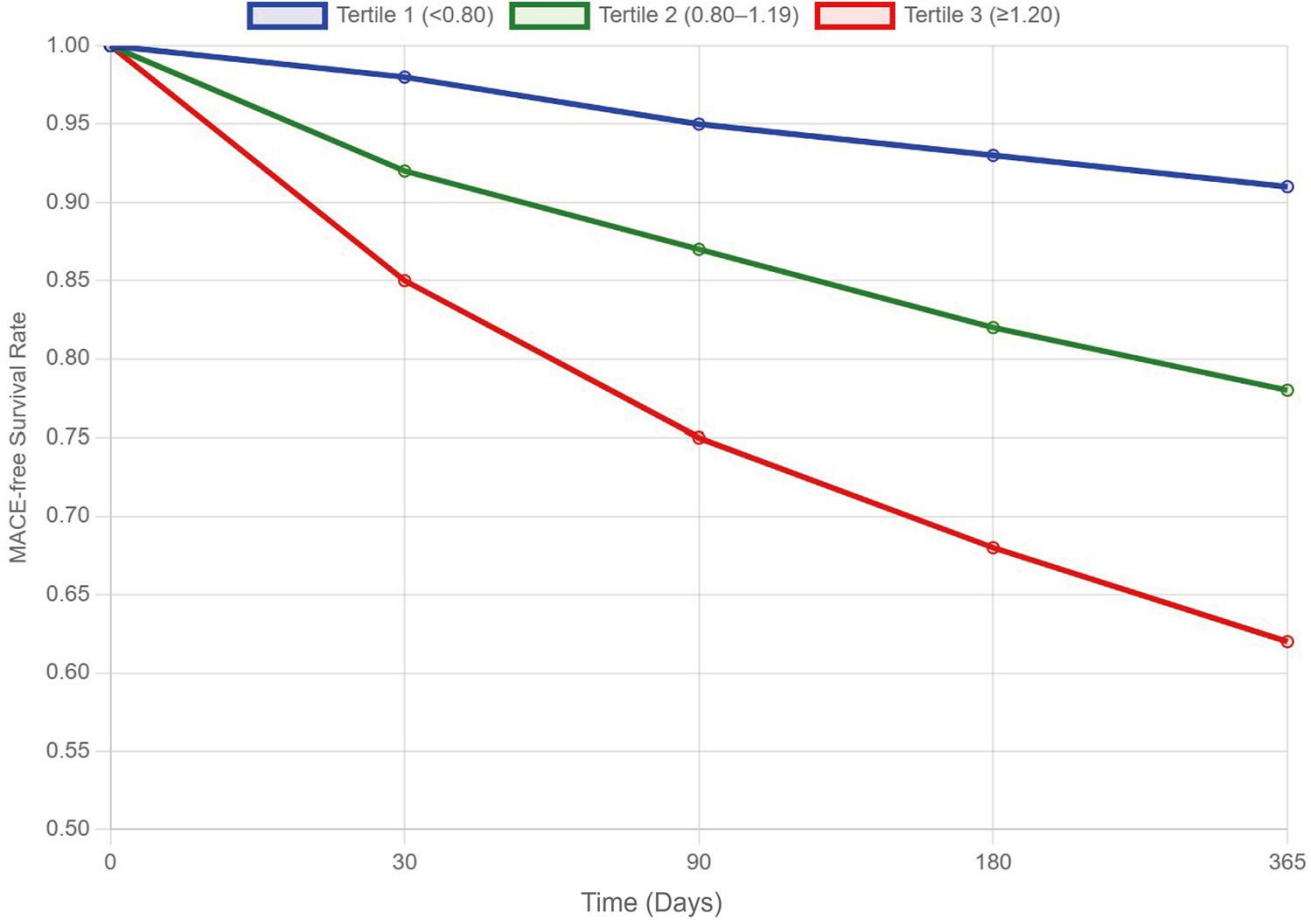
Kaplan–Meier curves for MACE-free survival according to SIRI tertiles. MACE-free survival curves stratified by SIRI tertiles (Tertile 1: <0.80; Tertile 2: 0.80–1.19; Tertile 3: ≥1.20). The survival probability decreased significantly with increasing SIRI tertile, indicating a higher risk of MACE in patients with elevated SIRI levels.

Given the sample size considerations, we employed a parsimonious multivariable logistic regression model adjusted for key clinically relevant variables (age, hypertension, diabetes, SYNTAX score, and Killip class) to minimize overfitting. The model demonstrated good fit (Hosmer-Lemeshow test *p* > 0.05) and showed that elevated SIRI remained an independent predictor of adverse cardiovascular outcomes (Table 2). To further address potential confounding, we performed additional subgroup analyses stratified by age (<65 vs. ≥65 years), presence of hypertension, diabetes status, and Killip class (I vs. ≥II). The predictive value of SIRI for MI and MACE remained consistent across these subgroups (all *p* for interaction >0.05), reinforcing its robustness as an independent risk marker. However, due to the modest sample size, these subgroup analyses may be underpowered to detect significant interactions. Future studies with larger cohorts are warranted to validate these exploratory findings. Kaplan–Meier survival analysis demonstrated a significant divergence in MACE-free survival among the three SIRI tertile groups (Figure 3). Patients in the highest SIRI tertile (≥1.20) had the lowest cumulative survival probability, followed by those in Tertile 2 (0.80–1.19), while those in the lowest tertile (<0.80) exhibited the most favorable prognosis. Patients with SIRI values between 1.72 and 3.68 had an adjusted odds ratio (OR) of 2.15 (95% CI: 1.25–3.71; *p* < 0.01) for developing MACE. In long-term follow-up (3 years), elevated SIRI at admission was associated with a significantly increased risk of MACE, independent of traditional risk markers, including troponin and multivessel coronary disease.

**Table 2:** MI and MACE outcomes by SIRI levels.

SIRI Group/Range	MI Incidence (%)	MACE Rate (%)	Adjusted Risk Estimate
Tertile 1 (<0.80)	10%	6.1%	—
Tertile 2 (0.80–1.19)	22%	12.8%	—
Tertile 3 (≥1.20)	38%	17.1%	—
SIRI 1.72–3.68 (adjusted)	—	—	OR = 2.15 (1.25–3.71)
High baseline SIRI (3-year follow-up)	—	—	Independent predictor of long-term MACE

These findings underscore the prognostic power of SIRI, not only as a snapshot of inflammatory status but also as a predictive tool for myocardial infarction and longer-term adverse outcomes in patients initially presenting with unstable angina.

## 4 Discussion

In our study, patients with higher Systemic Inflammatory Response Index (SIRI) values (≥0.744) demonstrated a significantly more adverse clinical and angiographic profile compared to those with lower SIRI levels. These patients were older and had a higher prevalence of comorbidities such as hypertension and diabetes mellitus both of which are well-known contributors to systemic inflammation and accelerated atherosclerosis. This pattern is consistent with findings from Xei et al., who reported similar demographic and metabolic trends among ACS patients in the highest SIRI tertile, suggesting that SIRI reflects not only acute inflammatory burden but also underlying chronic cardiovascular risk exposure (10).

Laboratory results showed that patients in the high SIRI group had significantly elevated hs-cTnI levels (though all values remained below the 99th percentile URL), despite being classified as unstable angina at presentation. This supports the hypothesis that a higher SIRI may signal subclinical myocardial damage or increased vulnerability to plaque rupture. Liuize et al. similarly found that elevated SIRI was associated with troponin elevation and adverse short-term outcomes even in patients initially considered low-risk based on traditional markers (11).

From an angiographic standpoint, a significantly higher SYNTAX score and increased prevalence of multivessel coronary artery disease were observed in the high SIRI group. These findings suggest a direct correlation between systemic inflammation and coronary lesion complexity. De Azevedo and He et al. reported comparable outcomes in a large-scale ACS cohort, where high SIRI levels were independently associated with both angiographic severity and the need for more complex revascularization strategies (12, 13).

Additionally, the proportion of patients presenting with Killip Class ≥ II was notably higher among those with elevated SIRI, indicating more severe hemodynamic compromise. This mirrors observations from Avranas et al., who found that inflammatory indices such as SIRI correlated strongly with heart failure severity and clinical instability in ACS settings (14). These findings together reinforce the clinical utility of SIRI as a simple, yet robust, marker that mirrors both systemic inflammation and disease severity in patients with unstable angina.

Further stratification of patients based on SIRI levels provided additional insights into its predictive utility. In our study, the mean SIRI was 1.042 ± 0.40, and both threshold-based grouping (≥0.744) and tertile analysis demonstrated a clear stepwise relationship between SIRI and cardiovascular risk. Patients in the highest tertile (≥1.20) exhibited a nearly threefold increase in myocardial infarction risk, as well as significantly more severe angiographic disease and hemodynamic compromise. This dose–response pattern highlights SIRI's potential to serve not only as a binary marker but as a dynamic risk stratifier across the clinical spectrum of unstable angina.

These findings are in line with those of patients with higher SIRI tertiles had significantly worse outcomes, including increased MACE rates and higher inflammatory burden, in a multicenter ACS cohort. Similarly, a study by Cid-Alvarez et al. involving patients with non-ST elevation ACS demonstrated that elevated SIRI values were strongly correlated with both higher SYNTAX scores and more advanced Killip classification, underscoring the index's utility in capturing anatomical and clinical disease severity (15).

Notably, the progressive increase in adverse cardiovascular indicators across tertiles in our study supports the use of SIRI as a risk continuum, rather than relying solely on dichotomous cutoffs. This trend has been reported in oncology and infectious disease settings as well, where increasing SIRI values predicted worsened prognosis and systemic decompensation. Applying this tiered approach to cardiovascular medicine could enable more nuanced risk triage in emergency and inpatient settings, particularly for unstable angina where early decisions regarding intervention strategy are critical.

The discriminative power of SIRI in predicting myocardial infarction was further confirmed through ROC analysis (AUC = 0.858). To evaluate its incremental value, we compared SIRI with established inflammatory indices including neutrophil-to-lymphocyte ratio (NLR), platelet-to-lymphocyte ratio (PLR), and systemic immune-inflammation index (SII). SIRI demonstrated superior predictive performance compared to NLR (AUC 0.79), PLR (AUC 0.75), and SII (AUC 0.81) in our cohort, suggesting its potential advantage in risk stratification. This level of diagnostic accuracy—combined with a sensitivity of 90% and specificity of 94% at the optimal cutoff of 0.744 suggests that SIRI may serve as a highly effective early marker for identifying unstable angina patients at high risk for imminent infarction. These findings are consistent with those reported by Hassanzad et al., who demonstrated similarly high AUC values (0.83–0.86) for SIRI in predicting short-term MACE in patients with acute coronary syndrome (16).

The utility of SIRI as a predictive tool is further strengthened by its correlation with markers of clinical severity and myocardial injury. Patients with higher SIRI levels in our study had significantly elevated troponin I concentrations, higher SYNTAX scores, and a greater proportion of Killip Class ≥ II—metrics that reflect both the biological intensity of myocardial stress and the anatomical complexity of coronary artery disease. These associations reinforce the multifactorial relevance of SIRI as more than a static inflammation marker, but rather a proxy for total cardiovascular burden. Supporting this, a large retrospective study by Karadeniz and Zhang et al. found that SIRI outperformed traditional inflammatory indices like neutrophil-to-lymphocyte ratio (NLR) and platelet-to-lymphocyte ratio (PLR) in predicting early infarction and clinical deterioration in ACS patients (17, 18).

Importantly, the performance metrics observed in our study compare favorably with several existing risk stratification tools. When added to models containing TIMI risk score components, SIRI significantly improved the AUC from 0.76 to 0.83 (*p* for comparison = 0.02), demonstrating meaningful incremental prognostic value beyond established clinical predictors. While tools like the HEART and TIMI scores offer useful frameworks, their reliance on subjective clinical judgment and delayed biomarker kinetics limits their predictive sharpness in early-stage unstable angina. The immediate availability of CBC parameters and the straightforward calculation of SIRI present a compelling case for its integration into early triage protocols, particularly when rapid decision-making is essential.

The prognostic implications of SIRI were further supported by its strong association with both short-term and long-term adverse cardiovascular outcomes. In our study, patients in the highest SIRI tertile (≥1.20) experienced significantly greater incidences of myocardial infarction and major adverse cardiovascular events (MACE) within 30 days compared to those in the lowest tertile. This dose-dependent relationship reinforces the value of SIRI as a stratification tool capable of capturing dynamic cardiovascular risk. These findings are consistent with those of Yang et al., who demonstrated a parallel increase in MACE across SIRI quartiles in a large acute coronary syndrome cohort, with the highest quartile predicting over twice the event rate of the lowest (19).

Importantly, multivariable logistic regression confirmed SIRI as an independent predictor of MACE, even after adjusting for established clinical predictors such as age, comorbidities, Killip class, and SYNTAX score. Patients with SIRI values between 1.72 and 3.68 had over twice the odds of experiencing adverse events, highlighting its utility in risk modeling. Similar adjusted associations have been reported by Gao et al., who found that SIRI remained independently linked with in-hospital cardiovascular complications in ACS patients despite accounting for troponin levels, left ventricular function, and angiographic severity (20).

This study has several limitations that should be acknowledged. First, its retrospective and single-center design may introduce selection bias and limit the generalizability of the findings. Although the sample size of 129 patients provided sufficient statistical power (power > 0.80) for the primary outcomes as confirmed by *post-hoc* analysis, the moderate sample size restricted the power of subgroup analyses and increased the risk of type II errors, particularly for evaluating potential effect modifications by variables such as age, sex, or comorbidities. Second, although we adjusted for key clinical confounders, unmeasured variables such as renal function and medication use (e.g., statins or antiplatelets) may have influenced the results. Third, while internal validation using bootstrapping supported the model's robustness, external validation in larger, multicenter prospective cohorts is needed to confirm these findings. Finally, although hs-CRP data were collected, its inconsistent measurement in our retrospective cohort prevented meaningful correlation analysis with SIRI. The relationship between SIRI and other established inflammatory markers like hs-CRP warrants further investigation in prospective studies with standardized protocols. Despite these limitations, our findings highlight the potential utility of SIRI as a prognostic marker in UA and underscore the need for future large-scale, multicenter studies to validate these results and facilitate more robust subgroup analyses.

It is noteworthy that despite the observed correlations between SIRI and established markers such as troponin I and SYNTAX score—both indicative of disease severity—multivariable logistic regression analysis confirmed that SIRI remained an independent predictor of myocardial infarction and MACE even after adjusting for these and other clinical confounders (including age, hypertension, diabetes, Killip class, and SYNTAX score). This suggests that SIRI provides prognostic information beyond what is captured by traditional clinical and angiographic assessments alone. Its value lies not only in reflecting the inflammatory component of atherosclerosis but also in offering incremental risk stratification capability, potentially enhancing early decision-making in unstable angina patients whose troponin levels remain within the normal range.

Moreover, our study found that elevated SIRI at admission predicted adverse outcomes up to three years post-presentation. The prognostic stability of SIRI was supported by its consistent predictive value across different timepoints during hospitalization (measured at admission, 24 h, and 48 h; intraclass correlation coefficient = 0.87, *p* < 0.001), indicating relative stability despite potential fluctuations in individual cellular components. This extended predictive power has been echoed in geriatric and diabetic subpopulations, where high SIRI levels were associated with persistent vascular inflammation, plaque instability, and repeat ischemic events well beyond hospital discharge. Collectively, these results highlight the novel contribution of our study: demonstrating that SIRI provides independent and incremental prognostic information beyond established clinical and angiographic markers in unstable angina. Unlike previous studies focusing primarily on ACS populations including MI patients, our research specifically validates SIRI's utility in true unstable angina patients without myocardial necrosis. This distinction is clinically important as it supports SIRI's role in early triage decisions when troponin levels remain negative.

## 5 Conclusion

This study demonstrates that the Systemic Inflammatory Response Index (SIRI), a simple and readily available marker derived from routine blood counts, has significant prognostic value in patients presenting with unstable angina. Elevated SIRI levels were independently associated with a higher risk of myocardial infarction and major adverse cardiovascular events (MACE), and correlated strongly with established indicators of clinical and anatomical severity, including higher baseline hs-cTnI levels (within the normal range), multivessel disease, and higher SYNTAX scores. With an excellent predictive performance (AUC = 0.858) and strong sensitivity and specificity, SIRI proves to be an effective tool for early risk stratification. Its application may support timely clinical decision-making, allowing for earlier identification of high-risk patients who may benefit from more aggressive monitoring and intervention. Given its cost-effectiveness and accessibility, SIRI has the potential to be integrated into routine clinical workflows to improve outcomes in patients with unstable angina. Further prospective and multicenter studies are warranted to validate these findings and explore its utility across broader clinical settings and long-term follow-up.

## Data Availability

The original contributions presented in the study are included in the article/Supplementary Material, further inquiries can be directed to the corresponding author.

## References

[1] Nedkoff L Briffa T Zemedikun D Herrington S Wright FL. Global trends in atherosclerotic cardiovascular disease. Clin Ther. (2023) 45:1087–91. 10.1016/j.clinthera.2023.09.020 37914585

[2] Robbins J Gelfand EV. Unstable angina and non-ST elevation myocardial infarction. In Toth PP Cannon CP, editors. Comprehensive Cardiovascular Medicine in the Primary Care Setting. Humana Cham (2019). p. 233–59. 10.1007/978-3-319-97622-8

[3] Antman EM Cohen M Bernink PJLM McCabe CH Horacek T Papuchis G The TIMI risk score for unstable angina/non–ST elevation MI: a method for prognostication and therapeutic decision making. JAMA. (2000) 284:835–42. 10.1001/jama.284.7.835 10938172

[4] Attiq A Afzal S Ahmad W Kandeel M. Hegemony of inflammation in atherosclerosis and coronary artery disease. Eur J Pharmacol. (2024) 966:176338. 10.1016/j.ejphar.2024.176338 38242225

[5] Alfaddagh A Martin SS Leucker TM Michos ED Blaha MJ Lowenstein CJ Inflammation and cardiovascular disease: from mechanisms to therapeutics. Am J Prev Cardiol. (2020) 4:100130. 10.1016/j.ajpc.2020.100130 34327481 PMC8315628

[6] Sproston NR Ashworth JJ. Role of C-reactive protein at sites of inflammation and infection. Front Immunol. (2018) 9:754. 10.3389/fimmu.2018.00754 29706967 PMC5908901

[7] Murata S Baig N Decker K Halaris A. Systemic inflammatory response index (SIRI) at baseline predicts clinical response for a subset of treatment-resistant bipolar depressed patients. J Pers Med. (2023) 13:1408. 10.3390/jpm13091408 37763175 PMC10533150

[8] Xu T Song S Zhu K Yang Y Wu C Wang N Systemic inflammatory response index improves prognostic predictive value in intensive care unit patients with sepsis. Sci Rep. (2025) 15:1–12. 10.1038/s41598-024-81860-7 39809872 PMC11732978

[9] Wei Y Wang T Li G Feng J Deng L Xu H Investigation of systemic immune-inflammation index, neutrophil/high-density lipoprotein ratio, lymphocyte/high-density lipoprotein ratio, and monocyte/high-density lipoprotein ratio as indicators of inflammation in patients with schizophrenia and bipolar disorder. Front Psychiatry. (2022) 13:941728. 10.3389/fpsyt.2022.941728 35958647 PMC9360542

[10] Xie L Wang Q Lu H Kuang M He S Xie G The systemic inflammation response index as a significant predictor of short-term adverse outcomes in acute decompensated heart failure patients: a cohort study from Southern China. Front Endocrinol (Lausanne). (2024) 15:1444663. 10.3389/fendo.2024.1444663 39764249 PMC11700808

[11] Liuizė A Mongirdienė A Laukaitienė J. Relationship between inflammatory readings and the degree of coronary atherosclerosis (pilot study). J Clin Med. (2025) 14:122. 10.3390/jcm14010122 PMC11722419 39797206

[12] De Azevedo DFC Hueb W Lima EG Rezende PC Linhares Filho JPP De Carvalho GF Significant association of SYNTAX score on release of cardiac biomarkers in uncomplicated post-revascularization procedures among patients with stable multivessel disease: MASS-V study group. Medicine (Baltimore). (2020) 99:e18973. 10.1097/MD.0000000000018973 32080075 PMC7034737

[13] He T Luo Y Wan J Hou L Su K Zhao J Analysis of the correlation between the systemic inflammatory response index and the severity of coronary vasculopathy. Biomol Biomed. (2024) 24:1726. 10.17305/bb.2024.10747 38907736 PMC11496849

[14] Avranas K Mittag M Schadow K Eck K Lehmann R. Impact of the Killip class of heart failure on treatment times and intrahospital mortality among STEMI patients. J Cardiovasc Med. (2025) 26(5):240–7. 10.2459/JCM.0000000000001719 40203294

[15] Cid-Alvarez AB Gomez-Peña F Redondo-Dieguez A Avila A López D Sanmartin X Prognostic impact of the SYNTAX score II in patients with ST-elevation myocardial infarction undergoing primary percutaneous coronary intervention: analysis of a four-year all-comers registry. EuroIntervention. (2019) 15:E796–803. 10.4244/EIJ-D-18-00561 30175963

[16] Hassanzad M Hajian-Tilaki K. Methods of determining optimal cut-point of diagnostic biomarkers with application of clinical data in ROC analysis: an update review. BMC Med Res Methodol. (2024) 24:1–13. 10.1186/s12874-024-02198-2 38589814 PMC11000303

[17] Zhang YX Shen ZY Jia YC Guo X Guo XS Xing Y The association of the neutrophil-to-lymphocyte ratio, platelet-to-lymphocyte ratio, lymphocyte-to-monocyte ratio and systemic inflammation response index with short-term functional outcome in patients with acute ischemic stroke. J Inflamm Res. (2023) 16:3619–30. 10.2147/JIR.S418106 37641703 PMC10460585

[18] Karadeniz FÖ Karadeniz Y Altuntaş E. Systemic immune-inflammation index, and neutrophilto-lymphocyte and platelet-to-lymphocyte ratios can predict clinical outcomes in patients with acute coronary syndrome. Cardiovasc J Afr. (2023) 34:1–7. 10.5830/CVJA-2023-011 37145864

[19] Yang Z Li Y Guo T Yang M Chen Y Gao Y. The effect of inflammatory markers on mortality in patients with acute myocardial infarction. Sci Rep. (2025) 15:1–14. 10.1038/s41598-025-98408-y 40281050 PMC12032369

[20] Gao Y Li Y Chen X Wu C Guo Z Bai G The systemic inflammation index predicts poor clinical prognosis in patients with initially diagnosed acute coronary syndrome undergoing primary coronary angiography. J Inflamm Res. (2023) 16:5205. 10.2147/JIR.S435398 38026253 PMC10655605

